# Advances in Cytoplasmic Male Sterility in Sugar Beet from Mitochondrial Genome Structural Dynamics and Nuclear-Cytoplasmic Coordination

**DOI:** 10.3390/ijms262010175

**Published:** 2025-10-19

**Authors:** Weiting Zhong, Shuo Zhang, Xiaolin Gu, Yanghe Zhao, Zhiqiang Wu, Dali Liu, Wang Xing

**Affiliations:** 1 National Beet Medium-Term Gene Bank, Heilongjiang University, Harbin 150080, China; 2240105@s.hlju.edu.cn; 2 Shenzhen Branch, Guangdong Laboratory of Lingnan Modern Agriculture, Key Laboratory of Synthetic Biology, Ministry of Agriculture and Rural Affairs, Agricultural Genomics Institute at Shenzhen, Chinese Academy of Agricultural Sciences, Shenzhen 518120, China; szhang@webmail.hzau.edu.cn (S.Z.); guxl9523@163.com (X.G.); zyh15525162637@163.com (Y.Z.); wuzhiqiang@caas.cn (Z.W.); 3 College of Plant Science & Technology, Huazhong Agricultural University, Wuhan 430070, China; 4 China National Seed Group Co., Ltd., Sanya 572000, China

**Keywords:** sugar beet, cytoplasmic male sterility (CMS), organelle genome, mitochondria, gene editing

## Abstract

Sugar beet (*Beta vulgaris* L.) is a globally important sugar crop whose hybrid breeding system relies heavily on cytoplasmic male sterility (CMS) lines. Recent advances in sugar beet genomics, particularly the release of high-quality reference genomes and the characterization of organellar genomes, have provided a foundation for elucidating the molecular genetic mechanisms of CMS. Furthermore, innovations in gene editing technologies are enabling transformative functional studies in this field. The precise targeting of CMS-associated mitochondrial genes and nuclear restorer-of-fertility genes not only allows for direct investigation of theoretical models governing fertility regulation through nuclear–cytoplasmic interactions but also holds promise for the targeted development of sterile and restorer lines. This review systematically summarizes progresses in sugar beet genomics, the development of gene editing tools, and the current understanding of the molecular genetics of CMS and fertility restoration in sugar beet. Although challenges remain—such as efficient delivery of editing tools into mitochondria and coordinated editing of multiple genes—the integration of genomic and gene editing technologies is expected to accelerate multi-omics-guided dissection of CMS mechanisms. These advances will facilitate the precise design of high-yield, high-sugar, and stress-resistant sugar beet hybrids, thereby providing core scientific and technological support for the sustainable development of the global sugar industry.

## 1. Introduction

Sugar beet (*Beta vulgaris* L.), as the world’s second largest sugar crop after sugarcane, serves as a critical economic pillar in temperate regions. The high concentration of sucrose stored in its roots represents a major source of edible sugar, while its by-products (such as molasses and pulp) play important roles in the food, feed, and bioenergy industries. With continued global population growth and rising consumer demands, the need for sugar and sugar-derived products is steadily increasing, driving the sugar beet industry toward a higher yield, increased sugar content, enhanced stress resistance, and improved production efficiency. The exploitation of heterosis for developing high-yielding and superior hybrid cultivars has been demonstrated as a core strategy for enhancing productivity and competitiveness in sugar beet production. However, the crop’s obligate outcrossing nature and frequent interspecific hybridization have resulted in a complex genetic background, which poses significant challenges to precision breeding efforts [1]. Therefore, a deeper understanding of its genetic mechanisms, particularly the molecular basis of fertility control, is crucial for advancing sugar beet breeding technologies.

Male sterility refers to a phenomenon in sexual reproduction where the stamen fails to develop normally or produce viable pollen, while the pistil remains functional and capable of fertilization. This is a widespread occurrence across the plant kingdom [2]. In hybrid breeding systems, male sterile lines are used to control pollination, enabling efficient production of high-yielding hybrid varieties. To date, male sterility has been reported in 617 plant varieties or interspecific hybrids, spanning 43 families, 162 genera, and 320 species—including major crops such as maize (*Zea mays* L.), sorghum (*Sorghum bicolor* L.), rice (*Oryza sativa* L.), and rapeseed (*Brassica napus* L.) [3]. Male sterility is generally categorized into two major types: genic male sterility (GMS) and cytoplasmic male sterility (CMS), with the latter being of paramount importance to hybrid breeding in sugar beet. Consequently, this article concentrates on recent progress and applications related to CMS.

As the predominant sterility mechanism employed in plant hybrid breeding, CMS serves as the critical biological basis for large-scale hybrid seed production in sugar beet. Using a CMS line as the female parent and crossing it with a restorer line carrying the restorer-of-fertility (*Rf*) gene enables efficient production of F1 hybrid seeds. Meanwhile, male-sterile lines are maintained using a maintainer line for propagation. This classical “three-line” system for hybrid seed production has been extensively adopted in the sugar beet industry, significantly reducing the cost and labor associated with manual emasculation while ensuring high purity of hybrid seeds [4]. However, the complex genetic background of sugar beet poses a major constraint on deeper investigation and effective application of the CMS system. Therefore, a critical imperative is the precise delineation of its nuclear and organellar genomes to enable the comprehensive identification of CMS-related genetic elements and the clarification of their operative mechanisms.

Over the past decade, advances in sugar beet genomics have provided a robust foundation for analyzing the molecular mechanisms underlying fertility. Since the release of the first reference genome in 2014, rapid developments in sequencing technologies—such as PacBio long-read sequencing and Hi-C chromatin conformation capture—have enabled the assembly of higher-quality, chromosome-scale genomes (e.g., RefBeet-1.2.2) [5]. These improvements have opened new avenues for fine mapping of genes controlling key agronomic traits, including sugar content, disease resistance, root development, and bolting/flowering regulation. They have been particularly instrumental in creating opportunities to decipher the molecular genetics of sterility and fertility restoration. The continued integration of functional, comparative, and epigenetic genomics is steadily unraveling the complexity of these traits, yet conventional genetic methods face constraints in functional validation and breeding deployment. Here, gene editing technology, notably the rapidly evolving Clustered regularly interspaced Short Palindromic Repeats (CRISPR)/CRISPR-associated systems (Cas), provides a transformative approach for precisely investigating CMS-related gene functions and for the targeted creation of novel germplasm. Based on a systematic review of genomic discoveries and CMS mechanism research, this review will critically assess the demonstrated utility, potential, key challenges, and future trajectory of gene editing tools in advancing sugar beet CMS studies.

## 2. Advances in Sugar Beet Genomics Research

CMS is primarily caused by nucleo-cytoplasmic interactions—genetic incompatibilities between the nuclear genome and organellar genomes, such as those in mitochondria and chloroplasts. Within plant cells, mitochondria and chloroplasts are semi-autonomous organelles harboring their own genetic material. Their evolutionary origin is explained by the endosymbiotic theory: ancestral eukaryotic cells engulfed alphaproteobacteria and cyanobacteria, which later evolved into mitochondria and chloroplasts, respectively. Over hundreds of millions of years, these organelles underwent extensive gene loss and large-scale transfer of genetic material to the nuclear genome, resulting in highly reduced organellar genomes that retain only core genes essential for energy metabolism and other fundamental functions [6,7].

In sugar beet, studies of the nuclear genome have provided critical insights into its domestication history, genetic diversity, and the regulatory mechanisms of agronomically important traits. On the other hand, the mitochondrial genome—due to its structurally dynamic and recombination-prone nature and its close association with CMS—has become a major research focus aimed at improving stress tolerance and advancing hybrid breeding programs.

### 2.1. Advances in Nuclear Genome Research of Sugar Beet

The systematic characterization of the sugar beet nuclear genome serves as a foundation for probing its biological attributes, most notably the nucleo-cytoplasmic interplay in CMS. Following the first reference genome generated by Dohm et al. in 2014 [5], later refinements have yielded chromosome-level assemblies like RefBeet v1.2. Subsequent genomic sequencing of cultivated line EL10 [8], leaf beet (*B. vulgaris* var. *cicla* L.) [9], and wild relatives (*B. vulgaris* ssp. *maritima* and *Beta patula*) [10,11] has not only illuminated patterns of genetic divergence and evolutionary history via population genomics but also enabled precise, genome-wide identification of loci determining key agronomic traits [12].

Functional genomics studies of the nuclear genome directly inform the comprehension of the CMS restoration system. Investigations have clarified essential regulatory networks for stress resistance and development, ranging from analyses of key gene families [13,14] to post-transcriptional mechanisms in salt adaptation revealed by integrated mRNA and miRNA profiling [15]. Notably, high-quality genomes have facilitated quantitative trait loci (QTL) mapping and cloning efforts that identified specific resistance determinants, including a leaf spot-resistant bZIP transcription factor [16], a nematode tolerance locus [17], and a QTL for Fusarium yellows resistance [18]. These approaches provide a methodological paradigm for isolating nuclear *Rf* genes. The development of specialized annotation tools like “BeetRepeats” [19] for the sugar beet genome and PMGA [20] for plant mitochondrial genomes further supplies key resources for the systematic identification of nuclear candidate genes interacting with mitochondria.

In conclusion, nuclear genome research in sugar beet is transitioning from foundational assembly to functional characterization, a central goal of which is to define the roles and regulation of *Rf* genes in CMS. The integration of multi-omics data for fine-resolution analysis of nuclear-organellar interactions, coupled with the application of gene editing to precisely engineer new restorer germplasm, represents a crucial next step to advance both fundamental knowledge and breeding utility for CMS.

### 2.2. Advances in Chloroplast Genome Research in Sugar Beet

Although chloroplast genome studies are not directly linked to the phenotypic origin of CMS, chloroplasts, as carriers of maternal inheritance, play a significant role in controlling hybrid seed purity and analyzing germplasm evolution. The chloroplast, a key organelle for photosynthesis, possesses a genome that in most higher plants adopts a conserved quadripartite structure, consisting of a large single-copy (LSC) region, a small single-copy (SSC) region, and a pair of inverted repeats (IRs), although several atypical structural variations also exist [21,22,23]. Advances in high-throughput sequencing have facilitated the precise resolution and de novo assembly of the complete sugar beet chloroplast genome [24].

Pan-plastome analyses based on these data have revealed abundant single nucleotide polymorphisms (SNPs) and insertions/deletions (InDels) in sugar beet and its wild relatives, providing invaluable molecular markers for high-resolution phylogenetic studies and germplasm identification [25]. On the practical front, the maternal inheritance of chloroplasts effectively circumvents the risk of transgene flow via pollen, establishing plastid transformation in sugar beet as a promising and environmentally safe breeding strategy. This approach opens new avenues for introducing traits such as insect and disease resistance in the future [26]. Although the chloroplast genome itself is not the determinant of CMS, its study offers important insights into the patterns of cytoplasmic inheritance in sugar beet and contributes to the refinement of hybrid seed production systems.

### 2.3. Advances in Mitochondrial Genome Research in Sugar Beet

The mitochondrial genome is central to the occurrence and evolution of CMS in sugar beet. Unlike the structurally conserved chloroplast genome, the sugar beet mitochondrial genome exhibits remarkable plasticity. Its large size and complex repetitive sequences mediate frequent homologous recombination, leading to extensive genomic rearrangements, variations in gene content, and structural polymorphisms [27]. To date, 11 sugar beet mitochondrial genome assemblies have been deposited in NCBI, providing a rich resource for elucidating this structural diversity.

These structural variations are causally linked to CMS. Early studies using Owen-type CMS materials revealed that recombination between repetitive sequences drives structural changes [28], and comparative genomics between cultivated and wild beets has further confirmed substantial intra-species structural diversity [29]. At the molecular level, CMS is generally attributed to chimeric open reading frames (ORFs) generated by mitochondrial genomic rearrangements. To date, three major CMS types have been identified in sugar beet: Owen [4], E [30], and G [31] types, with the mitochondrial genome of the G-type sterile line fully sequenced [32]. The aberrant expression products of these chimeric ORFs disrupt normal pollen development, and their toxic effects can be suppressed by nuclear-encoded restorer-of-fertility (*Rf*) genes, forming the classic “genomic conflict” model of the CMS/Rf system. Genome-wide association studies have successfully mapped key genomic regions linked to CMS traits, laying a solid foundation for the eventual cloning of these chimeric ORFs [33].

However, the highly dynamic nature of the sugar beet mitochondrial genome presents unique and serious challenges for breeding applications. Based on this understanding, mitochondrial genomic information is being directly applied in breeding practice. First, frequent rearrangements mean that genetic markers associated with CMS may lose stability across different breeding materials or generations, complicating the development and broad application of molecular marker-assisted selection (MAS) systems based on a single reference genome [34]. Second, the mitochondrial and nuclear genomes engage in close and complex co-evolution. While the introduction of wild germplasm via introgressive hybridization can contribute beneficial cytoplasm, it may also lead to novel fertility issues due to nucleo-cytoplasmic incompatibility [35]. Most critically, the extreme complexity and recombinogenic activity of the mitochondrial genome make it exceptionally difficult to precisely validate the function of specific chimeric ORFs—let alone create or “correct” specific sterile cytoplasm—using conventional genetic approaches.

Thus, although current knowledge of the sugar beet mitochondrial genome has laid the groundwork for its utilization in breeding, its inherent biological complexity constitutes a key bottleneck in translating this knowledge into technological breakthroughs. This very challenge underscores the urgency and great potential of developing next-generation gene-editing technologies capable of directly and precisely manipulating the mitochondrial genome.

## 3. Advances in Cytoplasmic Male Sterility Research in Sugar Beet

**Figure 1:**
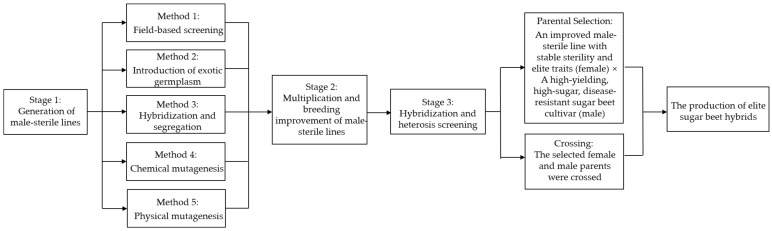
Process for Generating Elite Hybrids in Sugar Beet.

Commercial sugar beet varieties are almost exclusively hybrids, with their production dependent entirely on the exploitation of CMS [36]. The breakthrough application of CMS technology has fundamentally transformed sugar beet breeding. By leveraging nucleo-cytoplasmic interactions—specifically those between the mitochondrial and nuclear genomes—this system enables precise inhibition of pollen development, providing an efficient and cost-effective solution for large-scale hybrid seed production. Since the initial discovery of S-type CMS in sugar beet by Owen, the three-line breeding system based on CMS has become the mainstream approach for hybrid breeding in this crop [4]. Today, all commercial sugar beet varieties are hybrids utilizing CMS [1]. The use of CMS lines for hybrid seed production significantly enhances breeding efficiency, reduces costs, and strengthens heterosis. Male-sterile plants are commonly obtained through this protocol [37] (Figure 1). A deeper understanding of the molecular mechanisms underlying the formation and restoration of CMS in sugar beet is critical to creating novel sterile lines and developing high-performance hybrid cultivars with robust hybrid vigor.

### 3.1. Discovery and Types of CMS in Sugar Beet

**Figure 2:**
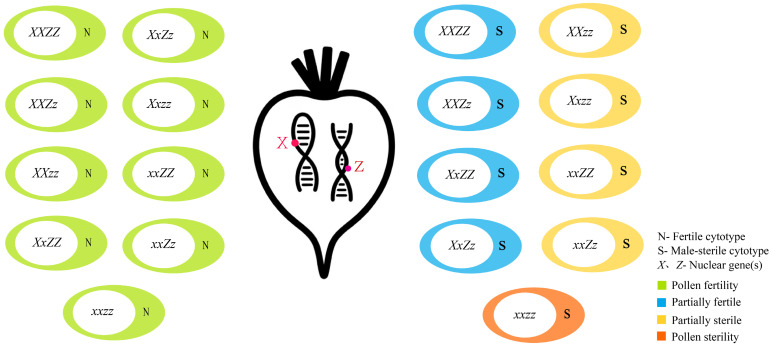
Categories of Cytoplasmic Male Sterility in Sugar Beet Mediated by Nuclear-Cytoplasmic Interactions.

Among the two main types of male sterility in sugar beet, CMS plays the predominant role in hybrid breeding. In 1945, Forrest V. Owen first identified a CMS plant from the sugar beet variety ‘US-1’, which was defined as the Owen-type CMS, initiating the era of CMS-based hybrid breeding in sugar beet [4]. Currently, approximately one-third of cultivated sugar beets carry Owen-type CMS, reflecting its broad adoption in hybrid programs. Owen proposed a genetic model involving the cytoplasmic gene *S* and two nuclear *Rf* genes, *X* and *Z*, located on chromosomes 3 and 4, respectively, to explain the inheritance of CMS in sugar beet [4]. In individuals heterozygous for chromosomal structural variations—such as reciprocal translocations or inversions—two types of gametes are produced: semi-sterile and semi-fertile. The former are nonviable due to genetic imbalance caused by chromosomal deletions or duplications, while the latter remain functional and capable of normal fertilization owing to their balanced genetic content [4]. When the cytoplasm is of the S-type, plants with the nuclear genotype *xxzz* exhibit complete male sterility, whereas those carrying either the dominant *X* or *Z* allele show partial sterility or semi-fertility. In contrast, all genotypes are fully fertile under N-type cytoplasm [4] (Figure 2).

Additional CMS germplasm types have also been identified through various studies. Furthermore, other studies have identified distinct CMS germplasm types. These include I-12CMS (3) from wild beets in Pakistan [38] and *G*CMS from the cytoplasm of wild sea beets in France [39], respectively. In investigations of Chinese sugar beet genetic resources, Cheng et al. [40] observed differences in variable number tandem repeat (VNTR) copy numbers between CMS and maintainer lines, which can be used to distinguish cytoplasmic types. Similarly, Nishizawa et al. [41] identified four distinct tandem repeat loci within the mitochondrial genome of sugar beet and developed four primer sets (TR1–TR4) for precise cytoplasmic typing. TR1, located within the rrn26 repeat family, consists of 32-bp units flanked by 7-bp direct repeats. Its copy number was significantly higher in the normal cytoplasm (13 copies) compared to two male-sterile cytoplasms (4–5 copies), suggesting a potential association with cytoplasmic male sterility. TR2 was highly conserved with 3 copies across all genotypes. TR3 exhibited a reduced copy number in the male-sterile lines, while TR4 showed no consistent variation pattern. These findings reveal the presence of minisatellite-like VNTRs in plant mitochondrial genomes, with TR1 polymorphism providing new insights into the molecular basis of cytoplasmic male sterility. In CMS-characterized plants, structural alterations—particularly within the vascular tissues of the stamen filament and the anther—have been associated with microspore sterility [42]. CMS is now extensively employed in commercial hybrid breeding across multiple crops, enabling the production of hybrid seeds with purity exceeding 99.9%, confirming that male-sterile plants serve as reliable maternal parents in hybrid seed production systems [43]. Many commercial sugar beet hybrids are produced via a three-way crossing scheme: a male-sterile F1 plant, derived from a cross between a CMS line and an unrelated maintainer line (to ensure the sterility trait), is pollinated by a fertile pollinator line. This approach ensures the maintenance of male sterility in the seed parent while facilitating large-scale hybrid seed production [1].

### 3.2. Mechanisms of Major CMS Types in Sugar Beet

Mitochondria, as semi-autonomous organelles with their own genetic material, may harbor selfish genetic elements. These are defined as “genetic units that evolve by enhancing their own transmission within the host genome, while being neutral or detrimental to the overall fitness of the organism” [44]. One class of such mitochondrial selfish genes is closely linked to the maternal inheritance of mitochondrial DNA: in bisexual flowering plants, these genes induce male sterility, thereby reallocating resources originally dedicated to male gamete production toward enhanced female gamete formation and somatic maintenance, ultimately gaining a transmission advantage [45]. This evolutionary drive provides a theoretical basis for the origin of mitochondrial-encoded genes that induce male sterility—a phenomenon defined as CMS [46]. Extensive studies on enzyme activities and structural analyses of mitochondrial DNA have established the mitochondrion as the key organelle responsible for pollen phenotypic alterations in CMS plants [47]. The Owen-type CMS mitochondria are found across all types of cultivated beet, including sugar beet [48], garden beet [49], fodder beet [50], and leaf beet [51].

Plant male sterility is classified into four primary molecular mechanisms: disturbed energy metabolism, retrograde regulation, aberrant programmed cell death, and cytotoxic effects [52]. CMS in sugar beet represents a core genetic system for exploiting heterosis and operates mainly through disrupted energy metabolism. This condition is directly linked to abnormal recombination of the mitochondrial genome and dysregulated interactions with the nuclear genome [47]. In the tapetum of CMS plants, a reduced number of mitochondria has been observed, resulting in lower ATP production [53]. Recombination events in mtDNA may generate chimeric open reading frames encoding transmembrane proteins, which can alter mitochondrial inner membrane permeability and disrupt the electrochemical potential [54]. DNA methylation exerted a negative regulatory effect, suppressing the expression of 75% of the related genes. This suggests that hypermethylation may contribute to pollen sterility in sugar beet by disrupting the normal function of fertility-related pathways [55].

### 3.3. Mitochondrial Genes Associated with Cytoplasmic Male Sterility in Sugar Beet

**Figure 3:**
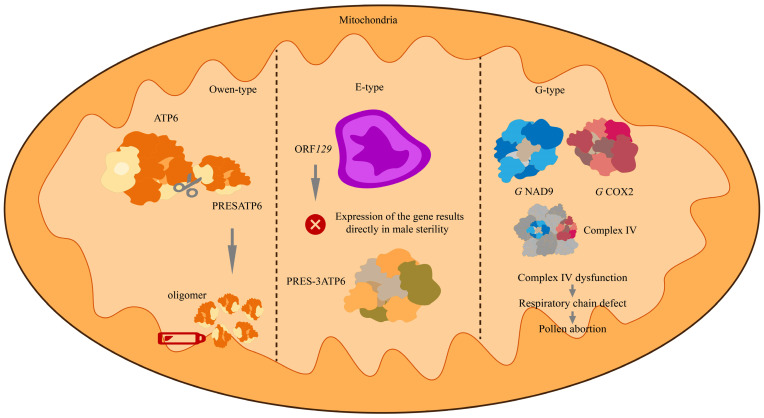
Molecular Mechanisms Underlying Three Major CMS Types in Sugar Beet. Owen-type: The N-terminal extension (*preSatp6*) of the mitochondrial *atp6* ORF is translated into stable homooligomers that anchor to the mitochondrial membrane, disrupting the energy metabolism essential for pollen development and ultimately leading to pollen sterility. E-type: The product of *orf129* localizes to both the mitochondrial membrane and matrix. Its transgenically expressed protein has been confirmed to induce male sterility. This type also encodes an N-terminal extension, *preS-3atp6*, through its protein abundance remains low in flower buds. G-type: Structural modifications occur in two respiratory chain complex subunits: NAD9 and COX2. The NAD9 subunit features a C-terminal extension, while the COX2 subunit contains a truncated C-terminus. These alterations disrupt the functional of complex IV and contribute to CMS phenotype.

Three major mitochondrial types that induce CMS have been identified in sugar beet, each characterized by distinct molecular alterations in mitochondrial genes or ORFs associated with the CMS phenotype (Figure 3).

Several candidate genes have been implicated in CMS in sugar beet. In the wild beet I-12CMS (3) cytoplasm, the mitochondrial gene *orf129* encodes a unique 12 kDa protein, ORF129, which localizes to mitochondria in flowers, roots, and leaves, specifically within the mitochondrial matrix and loosely associated with the inner membrane. Transgenic expression of *orf129* fused to a mitochondrial targeting sequence under the control of an anther-specific promoter in tobacco successfully induced pollen sterility. Studies demonstrated that mitochondrial import of ORF129 is essential for disrupting pollen development, leading to microspore degradation after the tetrad stage and abnormal persistence of the tapetum. Although fertility restoration does not affect ORF129 accumulation, this protein has been unequivocally identified as the direct causal agent of this type of cytoplasmic male sterility, providing important insights into CMS mechanisms and hybrid breeding applications [30].

In the G-type CMS line of wild beet, a point mutation occurs in the mitochondrial gene *cox2*, where the 253rd codon (TTA) is converted to a stop codon (TGA). This leads to premature translation termination, resulting in a truncated COX2 protein lacking eight amino acids at the C-terminus. Although this truncated COX2 protein is still incorporated into cytochrome c oxidase (Complex IV), its enzymatic activity is reduced by approximately 50%, and the stability of the complex is impaired, as evidenced by its failure to be effectively detected in native electrophoresis. While vegetative growth remains unaffected, pollen development is likely disrupted due to compromised respiratory chain efficiency. Concurrently, the expression of alternative oxidase is upregulated, possibly to partially compensate for the energy deficiency. This study indicates that the point mutation in the cox2 gene, by directly impairing the function of mitochondrial Complex IV, is likely a key factor contributing to G-type cytoplasmic male sterility [32].

In Owen-type CMS of sugar beet, the 5′ leader sequence of the *atp6* gene, designated preSatp6, is identified as a 387-amino-acid ORF. It is co-transcribed with the downstream atp6 core region and translated into a unique 35 kDa protein, preSATP6. This highly hydrophobic protein stably accumulates in the mitochondrial membranes of male-sterile lines and assembles into an approximately 200 kDa homomeric complex, which is absent in fertile mitochondria. Expression of preSATP6 is not influenced by different nuclear backgrounds or fertility-restorer genes, and its accumulation correlates strongly with pollen sterility. Although the precise molecular mechanism remains unclear, as a characteristic mitochondrial protein in Owen-type CMS, preSATP6 is suggested to disrupt mitochondrial membrane integrity and energy metabolism, thereby leading to the failure of pollen development [56].

### 3.4. Research and Application Advances in Fertility Restorer Genes in Sugar Beet

CMS can be suppressed by *Rf* genes, thereby restoring pollen fertility in carrier germplasms [57]. One or more *Rf* genes may exist within a species’ genome [58]. For instance, in CMS-T maize, two *Rf* genes—*Rf1* (or a functionally equivalent gene) and *Rf2*—are required to restore fertility [57]. Although fertility-restoring alleles can suppress the expression of cytoplasmic male sterility, it is often difficult to phenotypically distinguish plants carrying different *Rf* genotypes (e.g., heterozygous *Rfrf* vs. homozygous dominant *RfR*f) in conventional breeding programs. Therefore, elucidating the molecular basis that discriminates among *Rf* alleles is essential for the efficient selection of CMS lines (used as female parents) and restorer lines (used as male parents) in hybrid seed production [59].

In some cases, the genetics of fertility restoration in sugar beet are complex. Two restorer genes, designated X (*Rf1*) and Z (*Rf2*), have been identified in B. *vulgaris*, with the latter having minimal contribution to fertility restoration [57]. *Rf1* constitutes a complex gene cluster consisting of multiple copies of *Oma1*-like genes. These members, collectively referred to as *RF-Oma1*, are non-canonical paralogs and coexist with another canonical *Oma1* ortholog in the sugar beet genome [60]. Based on the biochemical properties of their encoded proteins, the cluster can be divided into two classes: one capable of binding CMS-associated mitochondrial proteins, and the other lacking this function. This specific binding is crucial for fertility restoration, as it alters the higher-order structure of the CMS protein complex [61].

Notably, the *Rf1* locus exhibits high polymorphism in both copy number and nucleotide sequence. This molecular diversity is directly associated with allelic phenotypic variation, with dominant, semi-dominant, subdominant, and recessive alleles having been identified, indicating that *Rf1* is a classic multi-allelic locus [62]. In transgenic experiments, one of the *RF-Oma1* genes was shown to enhance pollen fertility [61]. When this specific *RF-Oma1* copy was expressed in suspension cells of CMS beet, its translation product interacted with preSATP6, resulting in a novel 200 kDa protein complex. In contrast, *RF-Oma1* derived from a recessive *Rf1* allele lacked this activity [63].

Fertility restoration is fundamentally a nuclear genome-driven compensatory response to mitochondrial dysfunction. Kitazaki et al. revealed a unique mechanism in sugar beet involving the nuclear-encoded, non-PPR restorer gene *BvORF20*. The *BvORF20-preSATP6* complex was also detected in the anthers of fertility-restored beet plants [61]. The major restorer gene *Rf1* encodes a PPR protein that coordinates a dynamic regulatory network: it not only directly binds *orf20* mRNA to mediate C→U RNA editing but also synergistically reestablishes cellular homeostasis through multiple pathways, including recruiting the RNA editosome component MORF8, engaging the demethylase ROS1, and inhibiting the pro-apoptotic factor BAG6 [64]. Molecular marker technologies developed based on these findings have been applied in breeding. For instance, a KASP marker designed for the *Rf1* haplotype Hap-3b achieves a genotyping accuracy of 99.2% [35], while hypomethylated regions within the *Rf1* promoter serve as epigenetic markers for predicting restoration efficiency (r = 0.87) [46].

The expression of nucleo-cytoplasmic interactions can vary significantly with environmental conditions. For example, *Arabidopsis* nuclear substitution lines exhibit differential adaptive responses under varying environmental settings [65]. *Rf1* serves as the primary genetic determinant controlling the expression of Owen-type CMS. Arakawa et al. investigated the molecular function of *Rf1* and demonstrated that loss of its ability to bind to the CMS-related protein leads to sterility [66]. It was further proposed that other genes such as Z may contribute to thermosensitive male sterility.

## 4. Summary and Prospects

CMS in sugar beet is a fundamental biological system for hybrid breeding, and the depth of its research and breadth of its applications are directly linked to the sustainable development of the sugar beet industry. In recent years, high-quality assembly and annotation of both nuclear and organellar genomes—particularly insights into the structural diversity of the mitochondrial genome and the relationship between its recombination mechanisms and CMS—have provided a solid foundation for elucidating the molecular basis of cytoplasmic male sterility. High-resolution nuclear genome assemblies have not only revealed the domestication history and genetic diversity of sugar beet but have also enabled the precise identification of key nuclear factors for CMS, particularly the *Rf* genes. Integrated multi-omics studies have further established that the essence of CMS lies in “cytonuclear genomic conflict.”

The development of gene editing technology has provided transformative tools for CMS research in sugar beet. Plant mitochondrial genome editing represents an emerging frontier in agricultural biotechnology, aiming primarily to decipher gene function and develop novel germplasm through precise manipulation of mitochondrial DNA (mtDNA). Current mainstream gene editing technologies include zinc finger nucleases (ZFNs), transcription activator-like effector nucleases (TALENs), and the CRISPR/Cas system. Since its introduction, CRISPR/Cas9 has emerged as a third-generation gene editing technology due to its ease of design, cost effectiveness, and high efficiency, making it a powerful tool for genomic modification [67].

**Figure 4:**
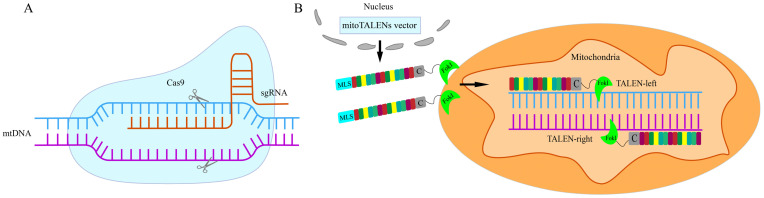
Schematic diagram of CRISPR/Cas9 and mitoTALENs technology. (**A**) CRISPR/Cas9 system uses a designed single-guide RNA (sgRNA) to precisely direct the Cas9 nuclease to recognize and bind specific DNA target sequences, where it induces a DSB. The double-membrane structure and negatively charged matrix of mitochondria hinder the delivery of exogenous editing components into the organelle. Furthermore, DSBs activate intrinsic cellular DNA repair mechanisms, thereby limiting the application of CRISPR/Cas9 in mitochondria. (**B**) MitoTALENs are engineered by replacing the N-terminal nuclear localization signal (NLS) of conventional TALENs with a mitochondrial targeting signal (MTS). Through nuclear genetic transformation, the T-DNA carrying the mitoTALENs construct is integrated into the nuclear genome of the host cell. The resulting MLS-TALE-FokI fusion protein is expressed in the cytoplasm and subsequently imported into mitochondria via the MTS. Within the organelle, the TALE-FokI fusion protein binds and edits its target gene.

However, mitochondrial genome editing faces unique challenges. Although the CRISPR/Cas9 system has been applied in mitochondrial genome editing, its reliability has been questioned due to insufficient experimental validation [68] (Figure 4A). In summary, while the application of CRISPR/Cas9 for mitochondrial genome editing is technically possible, current editing efficiency remains low, and the system is not yet broadly applicable. In contrast, the TALE-based mitoTALENs system has become the preferred platform for plant mitochondrial genome editing due to its high specificity and efficiency (Figure 4B). This technology has been widely used to investigate gene function and CMS mechanisms across various species, including the editing of *WA352* in rice [69], *orf137* in tomato [70], *atp6* in *Arabidopsis* [71], *nad9* in tobacco [72], and *orf138* in broccoli [73].

The mitoTALENs system has been successfully applied to achieve targeted knockout of plant mitochondrial genes, confirming the causal relationship between specific ORFs and CMS. Meanwhile, the emerging application of deaminase-based base editing systems in mitochondria shows great potential for precise single-nucleotide modifications, facilitating both mechanistic studies and the development of fertility-restoring lines. Although the genome of the sugar beet CMS-G sterile line has been sequenced, the specific sterility-inducing factor directly responsible for pollen sterility remains to be identified [74]. Current mitochondrial genome editing still faces challenges such as low efficiency, high off-target risks, and unstable delivery systems, particularly in crops with complex genomes like sugar beet, where widespread practical application has yet to be achieved.

Advances in pangenome studies and the completion of telomere-to-telomere (T2T) assemblies are propelling the discovery of sterility and restorer genes into a new phase [75]. Pangenomic resources provide comprehensive insights into structural variants (SVs) and gene content variation among individuals, offering a systematic identification of chimeric ORFs associated with CMS and nuclear restorer genes. The integration of long-read transcriptomics, epigenomic data, and deep learning-assisted annotation strategies will significantly enhance the accuracy of candidate gene prediction. Moreover, a meta-pangenome perspective will contribute to a holistic understanding of nucleo-cytoplasmic interactions [76]. These insights can be functionally validated through targeted CRISPR editing, facilitating a shift from conventional hybrid breeding toward precision-designed breeding. Future efforts should focus on improving the specificity and efficiency of editing tools, developing effective delivery systems for sugar beet mitochondria, and exploring the applicability of CRISPR-derived editors for mitochondrial genome modifications.

Precise editing of CMS-related genes offers the potential to develop fertility-controllable parent lines, thereby significantly enhancing hybrid seed production efficiency. Compared to traditional time-consuming backcross breeding, gene-editing technologies are expected to substantially shorten the breeding cycle and provide novel pathways for germplasm innovation. However, the widespread application of this approach still faces considerable challenges: precise editing of the mitochondrial genome remains technically demanding due to the lack of efficient targeted delivery systems; the risks of off-target effects and their potential impacts on cellular energy metabolism are not yet fully understood; furthermore, the complexity of multiplex gene editing, high R&D costs, and regulatory uncertainties collectively represent significant technical and economic barriers. Despite these challenges, with continued optimization of delivery systems, improved editing specificity, and advances in multiplex editing technologies, mitochondrial genome editing holds promise for elucidating plant energy metabolism mechanisms, deciphering nuclear-organellar interactions, and revealing the principles of environmental adaptation. Ultimately, it may serve as a core driver for global food security and sustainable agricultural development.

In conclusion, rapid advances in sugar beet genomics—particularly the development of high-quality reference genomes and the integration of multi-omics methodologies—have laid a solid foundation for systematically deciphering the molecular basis of CMS as a complex biological phenomenon. Although significant technical and economic hurdles remain in translating genomic knowledge into breeding practice, the deep integration of genomic information with precise editing capabilities will not only enhance our understanding of the molecular mechanisms underlying cytoplasmic male sterility in sugar beet but also accelerate the development of superior hybrid varieties with higher yield, increased sugar content, enhanced stress tolerance, and more efficient seed production systems. These innovations are poised to become key technological pillars for ensuring global sugar supply security and advancing the transition of the sugar beet industry toward a resource-efficient and environmentally sustainable future.

## Data Availability

No new data were created or analyzed in this study. Data sharing is not applicable to this article.

## Abbreviations

The following abbreviations are used in this manuscript:

CMS	Cytoplasmic male sterility
GMS	Genic male sterility
*Rf*	Restorer-of-fertility
CRISPR/Cas	Clustered regularly interspaced short palindromic repeats/CRISPR-associated systems
QTL	Quantitative trait loci
LSC	Large single-copy
SSC	Small single-copy
IRs	Inverted repeats
SNPs	Single nucleotide polymorphisms
InDels	Insertions/deletions
ORFs	Open reading frames
MAS	Marker-assisted selection
mtDNA	Mitochondrial DNA
ZFNs	Zinc finger nucleases
TALENs	Transcription activator-like effector nucleases
RVDs	Repeat variable diresidues
sgRNA	Single-guide RNA
MTS	Mitochondrial targeting signal
NLS	N-terminal nuclear localization signal
CBEs	Cytosine base editors
ABEs	Adenine base editors
VNTR	Variable number tandem repeat
